# Controlled rotation mechanism of DNA strand exchange by the Hin serine recombinase

**DOI:** 10.1038/srep23697

**Published:** 2016-04-01

**Authors:** Botao Xiao, Meghan M. McLean, Xianbin Lei, John F. Marko, Reid C. Johnson

**Affiliations:** 1 School of Physics, Huazhong University of Science and Technology, Wuhan, Hubei 430074, China; 2 Department of Physics and Astronomy, Northwestern University, Evanston IL 60208; 3 Key Laboratory of Molecular Biophysics of Ministry of Education, Huazhong University of Science and Technology, Wuhan, Hubei 430074, China; 4 Department of Biological Chemistry, David Geffen School of Medicine at UCLA, Los Angeles CA 90095-1737; 5 Department of Molecular Biosciences, Northwestern University, Evanston IL 60208

## Abstract

DNA strand exchange by serine recombinases has been proposed to occur by a large-scale rotation of halves of the recombinase tetramer. Here we provide the first direct physical evidence for the subunit rotation mechanism for the Hin serine invertase. Single-DNA looping assays using an activated mutant (Hin-H107Y) reveal specific synapses between two *hix* sites. Two-DNA “braiding” experiments, where separate DNA molecules carrying a single *hix* are interwound, show that Hin-H107Y cleaves both *hix* sites and mediates multi-step rotational relaxation of the interwinding. The variable numbers of rotations in the DNA braid experiments are in accord with data from bulk experiments that follow DNA topological changes accompanying recombination by the hyperactive enzyme. The relatively slow Hin rotation rates, combined with pauses, indicate considerable rotary friction between synapsed subunit pairs. A rotational pausing mechanism intrinsic to serine recombinases is likely to be crucial for DNA ligation and for preventing deleterious DNA rearrangements.

Site-specific recombinases catalyze a myriad of programmed DNA rearrangements that control processes such as gene assembly, DNA transposition, viral integration-excision, transcription, chromosome replication and segregation^1^. Most site-specific recombinases can be classified into two large families, named for their active site residues, that are unrelated in protein sequence and reaction mechanism^2^. Whereas tyrosine recombinases recombine DNA through a sequential single strand exchange mechanism involving a Holliday junction intermediate, serine recombinases that are the subject of this report recombine DNA through an intermediate containing double strand breaks. Site-specific recombination reactions are used extensively for genetic engineering and may have future utility for human therapy^3^,^4^,^5^,^6^.

A body of data supports a unique DNA exchange mechanism for serine recombinases whereby the tetrameric synaptic complex effectively forms a molecular swivel (see Fig. 1A)^7^,^8^,^9^. Initially, inactive dimers bind to specific recombination sites. Two DNA-bound dimers then associate and become remodeled into a chemically-active tetramer during formation of the synaptic complex. At the DNA cleavage step, each of the four subunits becomes covalently linked to the DNA via a serine ester bond to the 5′ phosphate, generating 2 bp staggered double strand breaks within the center of each recombination site. A 180° rotation of one of the newly synapsed pair of subunits (purple and gold in Fig. 1A) with their attached DNA strands about the other pair (blue and green) within the tetramer is believed to mediate DNA strand exchange.

Initial evidence for the subunit rotation mechanism for serine recombinases came from DNA topology studies on resolvases and DNA invertases. The primary products of these reactions on supercoiled circular DNA substrates are singly-catenated deletion circles (resolvase) and inversion on unknotted DNA circles with an accompanying ΔLk = +4 (DNA invertases)^10^,^11^,^12^,^13^. These products are consistent with a single 180° clockwise rotation of subunits within their respective higher order synaptic complex architectures. Even stronger topological support for the subunit rotation mechanism came from reaction conditions that encouraged or required multiple DNA exchanges prior to ligation (processive recombination reactions)^12^,^14^,^15^,^16^,^17^,^18^,^19^. The stereo structures of the resulting multiply catenated or knotted DNA products can only reasonably be accommodated by multiple subunit rotations between the DNA cleavage and ligation steps.

X-ray crystal structures of serine recombinases provide snapshots of different rotational conformers, with a relatively flat and exclusively aliphatic interface between rotating dimers (Fig. 1B)^20^,^21^,^22^,^23^. Relative to the first γδ resolvase structures solved, tetramers captured in the Gin resolvase and Sin invertase crystal structures have undergone rotations of 26° and 35–45°, respectively. Site-directed crosslinking experiments on the Hin serine DNA invertase have provided additional evidence for partial and complete subunit rotation within a tetramer^24^,^25^. Finally, observation of relaxation of two intertwined DNA duplexes tethered between a glass surface and magnetic bead by the Bxb1 serine integrase provides further evidence in favor of a subunit rotation reaction^26^.

Nevertheless, the subunit rotation mechanism has been controversial and indeed an alternative mechanism for DNA exchange within the serine recombinase tetramer structure has been proposed, based on a strand-passage mechanism similar to that of type-II topoisomerases^27^. Among the concerns raised are: (1) the danger of generating permanent chromosome breaks by a reaction where cleaved DNA ends are only held together by a slippery hydrophobic protein interface, (2) the potential for uncontrolled rotation causing massive DNA knotting, tangling, and loss of DNA supercoiling, and (3) the lack of precedent for such an extraordinary structural rearrangement within an otherwise stable protein complex.

To provide additional evidence for the subunit rotation mechanism and to address potential controls on the reaction, we have studied the Hin-H107Y serine DNA invertase mutant^28^ using a combination of bulk solution and single-DNA molecule approaches. Whereas the wild-type Hin recombinase requires the activity of the Fis/enhancer regulatory system and only catalyzes DNA inversion from a synaptic complex assembled at a supercoiled DNA branch^9^, Hin-H107Y can catalyze recombination on linear DNA molecules without accessory factors and through random collision synapsis pathways. The single amino acid change (histidine 107 to tyrosine) destabilizes the dimer and stabilizes the synaptic tetramer conformation that is active for DNA cleavage and exchange (Fig. 1C)^29^. As described below, these properties enable us to investigate biophysical parameters of the subunit rotation reaction by a serine DNA invertase in the absence of accessory regulatory elements. Our experiments provide strong evidence for a “controlled rotation” mechanism^30^,^31^ intrinsic to the recombinase tetramer structure that limits the number of revolutions per rotation event but does allow more than a half whole rotation.

## Results

### DNA recombination and cleavage by the Fis/enhancer-independent Hin-H107Y reaction

Bulk Hin-H107Y reactions were first studied to determine reaction parameters for the Fis/enhancer-independent mutant. Figure 2A,B show the results of recombination reactions catalyzed by Hin-H107Y without the accessory proteins Fis or HU on linear DNA substrates containing *hixL* recombination sites separated by 1636 bp. pMS634 (Fig. 2A, Supplementary Fig. 1) contains the *hixL* sites in inverted orientation to generate inversion of the intervening DNA segment, whereas pRJ858 (Fig. 2B, Supplementary Fig. 1) contains directly-oriented *hixL* sites and generates a 1634 bp deletion circle (linearized prior to electrophoresis) plus a 3.75 kb linear recombinant. Inversion and deletion reactions occur at nearly identical rates (Fig. 2C).

The nearly equivalent efficiencies of the inversion and deletion reactions by Hin-H107Y on linear substrates is consistent with a simple collision mechanism for site synapsis, in contrast with the Fis/enhancer-dependent wt-Hin reaction. Initial rates of Hin-H107Y inversion reactions performed on circular supercoiled pMS634 were 3-fold greater than those measured on the linearized DNA (Fig. 2C), probably because of the higher localized concentration of sites due to supercoiling^32^ and because supercoiling provides rotational energy for DNA exchange by subunit rotation when the synaptic complex traps a DNA branch (see below^24^). Evidence for Fis-independent recombination through random collision pathways of site synapsis on supercoiled plasmids is presented below.

Hin-H107Y catalyzed recombination is poor in the absence of divalent cations or polyamines, and Mg^2+^, Ca^2+^, or spermidine enhance the reaction similarly (Fig. 2D). Optimal Fis-independent Hin-H107Y recombination reaction conditions include 15% ethylene glycol (EG). In Fig. 2E increasing concentrations of EG were added to Hin-H107Y reactions containing EDTA on supercoiled pMS634. Some nicking is evident with no EG present, but with increasing amounts of EG, double strand cleavages at *hix* sites predominate. After a 15 min reaction with 30% EG and EDTA, <3% of the starting supercoiled DNA remained and >75% of the molecules were cleaved at both *hix* sites. Fig. 2F shows the kinetics of a Hin-H107Y cutting reaction on linear DNA with EDTA and 30% EG, the conditions employed in the single-DNA molecule experiments discussed below. Double strand cleavages at the two *hix* sites, which release the invertible segment from the flanking DNA ends upon denaturation of Hin by SDS, accumulate rapidly over the first 10 min. Only a small number of products attributable to a cleavage at only a single *hix* site are observed. The longer substrate pRJ2421, used in the single-DNA molecule synapsis assays, exhibited nearly identical reaction kinetics (Supplementary Fig. 2).

### Topological analysis of Hin-H107Y reactions: site synapsis and processivity of DNA exchange

We probed topological changes introduced by Hin-H107Y recombination into supercoiled plasmid substrates. To summarize, nearly all Fis/enhancer-activated Hin-H107Y DNA exchange reactions occur from synaptic complexes trapping a DNA branch (2 negative DNA nodes), indicative of an invertasome intermediate^14^,^33^. Unexpectedly, a large proportion of Hin-H107Y reactions in the absence of Fis proceed from a −2 DNA synapsis, but productive synapses via random collision pathways also occur. Hin-H107Y reactions that proceed through a −2 synaptic complex on supercoiled DNA tend to undergo variable numbers of processive subunit rotations prior to ligation. The processive nature of the subunit rotation reaction contrasts with the wild-type enzyme that usually undergoes only a single subunit rotation prior to ligation^12^,^14^. The increased processivity by Hin-H107Y may reflect the greater stability of the H107Y mutant tetramer^29^ combined with the trapped DNA supercoiling energy that is unique to the branched synaptic complex structure and will drive multiple subunit rotations^9^,^34^.

In Fig. 3A DNA knot profiles generated on pMS634 were resolved by gel electrophoresis. The enhancer in pMS634 is located about 700 bp from the closest *hix* site (Supplementary Fig. 1); therefore, multiple subunit exchanges can occur within a Fis/enhancer-containing invertasome structure without the torsional constraints of DNA windings that accompany multiple DNA exchanges, which occur with the native spacing where the enhancer is about 100 bp from a *hix* site^24^,^35^. Fis/enhancer-activated Hin-wt reactions on pMS634 generate a low number of knots (Fig. 3A, lane 3) because >95% of the reactions ligate after a single subunit exchange to generate an unknotted inversion product (see Fig. 3B)^12^,^14^. Less than 5% of the reactions undergo processive rotations whereby decreasing amounts of knotted molecules containing 3-, 4-, 5-, etc., nodes are generated. A trace amount of a 6-noded compound knot is also detectable after 5 min, which is created from two reactions each generating a trefoil knot. Fis/enhancer-activated reactions with Hin-H107Y give a similar, but more processive, pattern of knots on pMS634. Fifteen second reactions (lane 4) generate a nearly even distribution knots containing from 3- to >10-nodes. This profile is consistent with reactions initiating from a −2 branched invertasome intermediate and terminating after 2 to >9 clockwise subunit rotations (Fig. 3B) and potentially from random collision synapses.

Hin-H107Y reactions on pMS634 in the absence of Fis generate a similar profile of knotted molecules with products exceeding 10-nodes visible, even from short reaction times (Fig. 3A, lane 6 and 7). We believe this profile reflects primarily processive subunit rotation products formed from −2 synapses plus a lower number of products formed from random collision synapses. Evidence for the latter comes from restriction digestion of isolated trefoils, which showed that about a third were in the inverted orientation. These most likely originate from synapses trapping 4 (−) supercoils that have undergone a single clockwise subunit rotation (Fig. 3D). We note that because a substantial fraction of the unknotted products also contain inversions, Hin-H107Y reactions also likely proceed through “simple” synapses with no trapped DNA nodes, which will not result in an apparent linking number change upon one or more DNA exchanges.

pRJ862 is essentially identical to pMS634 except that it has a single base pair change within the core nucleotides of one of the *hixL* sites (Supplementary Fig. 1), which prevents DNA ligation in the recombinant (inverted) orientation. Ligation is therefore restricted to the parental orientation, requiring even numbers of DNA exchanges or 360° subunit rotations, thereby reducing the topological complexity of the products^14^. Initial Fis/enhancer-activated Hin-wt reactions (1 min incubation, Fig. 3A lane 9) generate primarily trefoils, the product of two 180° clockwise rotations (Fig. 3B). Very low amounts of pentafoils (product of four 180° clockwise rotations) and 6-noded compound knots are also present (Fig. 3B). Longer Hin-wt reactions (lane 10) generate a distributive pattern of knots reflecting multiple independent reactions. Large amounts of 3-, 6-, and 9-noded knots, which arise from one to three de novo reactions, respectively, are present along with products reflecting multiple reactions that independently generate knots with 3 and 5 nodes, e.g., knots with 8 and 11 nodes. Reactions by Hin-H107Y in the presence of Fis generate products primarily indicative of reactions proceeding through a −2 invertasome intermediate but that are more processive than Hin-wt. Short incubations (15 sec, lane 11) produce a series of knots containing odd number of nodes increasing by 2 (3-, 5-, 7- to at least 13-noded knots), reflecting up to at least 12 subunit rotations originating from a single initiation event prior to ligation. Most of the products in the longer Fis-activated reaction (lane 12), are consistent with single and multiple reactions through an invertasome intermediate. An estimate of the increased processivity of Hin-H107Y can be obtained from a comparison of the number of products generated from more than 2 rotations starting from the invertasome complex: 50% of H107Y reactions proceed beyond 2 rotations (lane 11), whereas <10% of Hin-wt products reflect more than 2 subunit rotations (lane 9).

Hin-H107Y reactions on pRJ862 in the absence of Fis generate a knotting profile that is consistent with a subset of reactions occurring by a random collision mechanism of synapsis in addition to a dominant pathway of processive exchanges from −2 synapses. The most prominent differences in the products from those by Hin-wt are the presence of 4- and 6-noded knots (Fig. 3A, lanes 13 and 14), which cannot form through a −2 synapsis on pRJ862 because of the inability to ligate over the mismatched core nucleotides. The 4-noded knot is generated from a random collision synapses that traps 3 (−) supercoils and undergoes 2 clockwise subunit rotations prior to ligation (Fig. 3C, Supplementary Table 1). The 6-noded twist knot, which migrates slightly faster than the compound 6-noded knot, can be generated by 2 counterclockwise rotations in a complex where 4 supercoil nodes are trapped and from 2 clockwise rotations in a complex where 5 supercoil nodes are trapped (Supplementary Table 1). The number of distributive reaction products is also reduced for Hin-H107Y reactions without Fis, as reflected by the lower number of 6-noded compound knots (compare lane 14 to lanes 12 or 10). We conclude that Hin-H107Y primarily initiates one reaction per substrate and supports some random collision synapses in the absence of the Fis/enhancer on supercoiled DNA.

### The DNA architectural properties of HU and Fis do not influence the Fis/enhancer-independent Hin-H107Y reaction

The HU nucleoid protein stimulates the Fis-activated Hin-wt reaction when the enhancer is in its native position close to one of the *hix* sites^36^,^37^ and was included in all of the above knotting reactions. The presence of HU has only a small stimulatory effect on Fis-independent Hin-H107Y inversion rates on pMS634 (not shown), and the only significant difference in the knot profile with pMS862 without HU is the complete absence of the compound 6-noded knot, the product of two independent reactions (Fig. 3A, lane 15). Likewise, the presence of a Fis mutant capable of binding and bending DNA, but missing the β-hairpin arms that specifically contact Hin^38^, does not change the knot profile as compared to the no Fis reaction (lane 16). Thus, the DNA bending proteins HU and Fis do not have a global conformational effect on the DNA that influences the otherwise Fis/enhancer-independent Hin reaction at protein levels that are optimal for the Hin-wt reaction.

### Synaptic complexes formed by Hin-H107Y on single-DNA molecules with two *hix* sites

**Figure 1:**
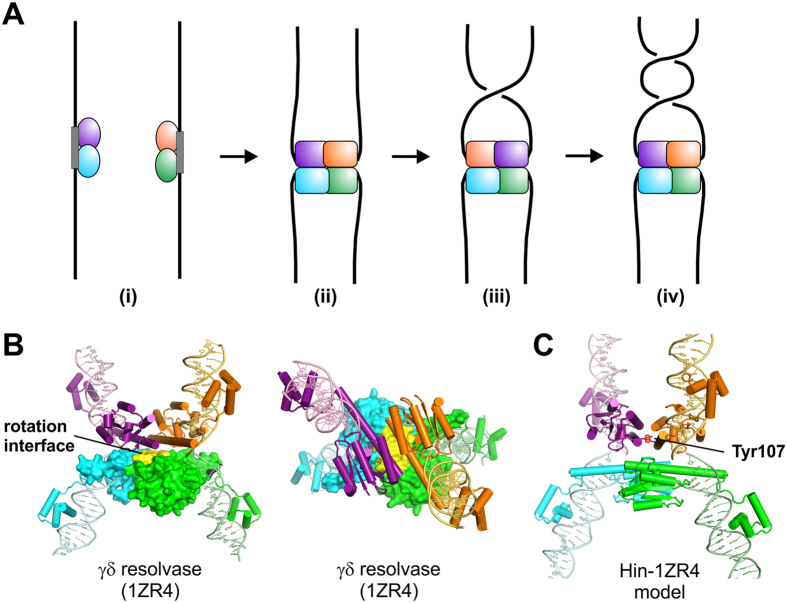
Recombination site synapsis and DNA exchange via subunit rotation by serine recombinases. (**A**) Schematic representation of the DNA exchange reaction mediated by serine recombinases in the absence of accessory controls, as is the case for the Hin-H107Y hyperactive mutant. (i) Two inactive dimers bound to specific recombination sites (*hix* sites in the case of Hin). (ii) Formation of the synaptic complex is coupled to remodeling of the dimers into a tetramer which then cleaves the four DNA strands generating serine phosphodiester bonds between each subunit and the 5′ DNA ends. (iii) Rotation of one pair of newly synapsed subunits (purple and gold) relative to the second pair (cyan and green) within the tetramer exchanges the DNA strands. (iv) Additional rotations may also occur prior to DNA ligation. **(B**) Two orientations of the γδ resolvase tetramer (PDB: 1ZR4) in a post DNA cleavage conformation as in (ii)^22^. A surface rendering of the bottom subunit pair is depicted with the aliphatic rotation interface colored yellow. **(C**) Model of the Hin-H107Y invertase tetramer based on 1ZR4^24^. The Tyr107 substitution (red) is believed to stabilize the tetramer through self-interactions between subunits of rotating dimers (purple and gold; cyan and green)^29^, in a manner similar to an arginine substitution at the analogous position of a hyperactive mutant of the Sin resolvase^21^.

We sought to obtain physical evidence for DNA synapses by Hin-H107Y, via detection of loop formation on single 9.5 kb DNA molecules containing two *hix* sites. Linearized pRJ2421, containing two *hixL* sites separated by 2368 bp, was attached to paramagnetic beads via a biotin-streptavidin bond at one end (Fig. 1A). The other end was attached to the cover glass of a flow cell, which was placed on a magnetic tweezers setup which allowed variable forces to be applied to the bead via positioning of permanent magnets. The force-extension response of a tether in PBS-BSA buffer was measured and used to find, validate and calibrate the tether as a single-DNA molecule^39^,^40^. When a single DNA was found, the PBS was replaced with Hin reaction buffer. Then 70 nM Hin-H107Y in the same buffer was flowed into the sample. Tether extension was continuously monitored (~100 measurements per second) while the force was held at 0.1 pN. During the period after the introduction of Hin-H107Y (typically within one hour), a sharp drop of tether extension was observed (Fig. 4D, between 25 and 26 min). The average loop size for pRJ2421 was 0.42 ± 0.04 μm from 5 experiments (Fig. 4F, left bar). This correlates well with the expected drop in length resulting from looping of the two *hix* sites that are spaced by 2.4 kb; a 2.4 kb segment of DNA under 0.1 pN force has an extension of 0.4 μm.

To demonstrate that the *hix* sites are required for this drop in tether extension, experiments with 6 kb control *hix*-free DNA (a PCR product made from the plasmid pFOS1) showed no such drops (Fig. 4E,F).

Following loop formation on pRJ2421, we increased the force to 2 pN to test the stability of the looped DNA; the loop was found to open (by observation of a large jump in extension), after a time lag ranging from 7 sec to 11 min, with an average loop-opening time of 16 ± 7 sec. The average DNA extension after loop opening was ~2.6 μm at 2 pN or ~1.6 μm at 0.1 pN, corresponding to the full length DNA. The fact that the DNA remained tethered indicates that the complexes had not been cleaved by Hin.

### Hin-H107Y mediates long-pause multi-step relaxation of catenated (braided) DNAs

Subunit rotation mediated by Hin-H107Y can be detected by direct observation of recombination between two torsionally unconstrained duplex DNAs wrapped around one another, or “braided”^26^ (Fig. 5A–F), so as to have an initial catenation number Ca (Ca corresponds to the number of toroidal DNA interwinds, relative to the unlinked state of two parallel DNAs). At a fixed force (0.5 pN in our experiments), the length of the braid depends on the linkage between the two DNAs (Fig. 5D); for Ca = 0, the braid has maximum length; as Ca is increased there is first a rapid drop in extension corresponding to “crossing” of the two DNAs, followed by a gradual drop as torque accumulates in the braid. If an active Hin synapse forms on a pair of braided DNAs (Fig. 5E), cleavage and rotation will lead to relaxation of the linkage between the two duplex DNAs, and therefore an increase of the extension of the braid. If either of the DNAs is broken, the braid immediately will lose all its stored Ca in one rapid step, which allows us to separate DNA breakage from Hin-mediated braid relaxation.

Two 5.9 kb DNA fragments derived from mMS502 containing a centrally located *hixL* site (Supplementary Fig. 1) were tethered to beads (Fig. 5B). The force-extension relationship and the extension-Ca number relationship at a fixed force of 0.5 pN were used to ascertain that a bead was tethered by two DNAs in PBS-BSA buffer (Fig. 5D); the calibration data allows braid extension to be related to Ca. The tether was then set to have Ca = −10 at a force of 0.5 pN. Finally, 70 nM Hin-H107Y in Hin reaction buffer was introduced into the flow cell, after which the tether extension was continuously monitored at 37 °C.

An example extension-time trace is illustrated in Fig. 5G. About 35 minutes after adding Hin-H107Y, an increase of 0.2 μm (from ~1.1 μm to ~1.3 μm) occurred (Fig. 5G). Second and third increases (steps) were subsequently observed at times of ~42 and ~51 min. These steps of extension indicate the stepwise relaxation of the braid, which can only be caused by the rotation of subunits in the synaptic complex. The mean time between addition of Hin in reaction buffer and the first relaxation was 1030 sec, corresponding to an apparent cleavage rate of (9.7 ± 2.5) × 10^−4^ s^−1^ (Fig. 5H, leftmost bar, N = 15).

In contrast, the extension remained stable for much longer periods in trials with control DNA, either the 6 kb fragment of pFOS1 or pNG1179 DNA (Fig. 5I shows a 200 minute experiment with no cleavage). The time to the first cleavage was much longer for control DNA than for *hix*-containing DNA, corresponding to much lower rates (Fig. 5H, second and third bars; apparent cleavage rates of 3.5 × 10^−5^ s^−1^ for pNG1179 and 6.4 × 10^−5^ s^−1^ for pFOS1; these events are likely simply random breakages of the DNA tethers). The *hix* sites are therefore essential for efficient DNA cleavage and braid relaxation.

We carried out a total of 15 Hin-H107Y braiding experiments, which led to observation of 41 relaxation steps. The number of full turns (360°, corresponding to two subunit exchanges) at each event was broadly distributed with a mean rotation number of 4.9 turns (Fig. 6A), suggestive of an exponential distribution with a mean larger than the number of turns initially put into the braid. There was no significant difference in the size of initial events (starting at a Ca of −10) relative to the last rotation event observed in each experiment (Supplementary Fig. 3). The pause time between events ranged from 24 s to 80 min, with a mean pause time of 22 ± 8 min.

### SDS-induced dissociation of complexes during the pause following the first rotation reaction

We tested whether the Hin synapse contained cleaved or religated DNA ends during the pauses between relaxation events. In five additional experiments, braids that had undergone the first Hin-H107Y-induced decatenation event were subjected to buffer replacement with reaction buffer plus 2% SDS. In all five trials, the bead was quickly lost (average time for bead release after addition of SDS was 11 ± 2 seconds), consistent with SDS-induced destabilization of the protein-protein interactions in a rotationally-stalled Hin protein complex containing unligated DNA ends. The bead release velocity after SDS denaturation-release was 12 ± 2 μm/s, six times that of the Hin H107Y-synapse rotation, and approximately the terminal velocity expected for a 2.8 μm-diameter “free” bead acted on by a 0.5 pN force^41^.

Control experiments where 2% SDS was added to the reaction buffer containing Hin-H107Y immediately prior to injection into the flow cell showed no change in braid catenation, demonstrating that this amount of SDS inactivates Hin but does not disturb the DNA tethers. We conclude that addition of SDS after the first relaxation event releases the two rotational halves of the DNA-cleaved Hin synaptic complex.

### The Hin-H107Y synapse limits the rate of relaxation of Ca

The step events themselves are characterized by relaxation velocities, which we measured by linear fitting. An average linear velocity of 1.8 ± 0.2 μm/s (Fig. 6B, leftmost bar) resulted, much slower than the “free” bead release velocity (~12 μm/s). This indicates that the Hin synapse limits the rate of removal of the braid Ca, in a manner similar to removal of supercoils by type IB and type IC topoisomerases and attributable to rotational friction in the synaptic complex^30^,^31^. This linear velocity corresponds to a mean rotational velocity of 28 ± 3 turns/s (Fig. 6C,D leftmost bar). A Gaussian distribution truncated at zero fits the rotational velocity distribution well, with a peak location of 27 turns/s and a standard deviation of 15 turns/s (Fig. 6C; Supplementary Fig. 4 shows a magnified view of individual step events).

### Post-decatenation behavior of complexes

In all cases where the braids were completely relaxed the bead length reflected X-shaped dual tethers, indicating that the synaptic complex had not dissociated. We attempted to re-catenate these braids by rotation of magnets. Five of the braids could be transiently catenated but soon reverted back to relaxed. Eight of the relaxed braids could not be re-catenated, indicating that the rotational surface underwent free rotation with cleaved DNA ends. One of the braids could be stably re-catenated, which could either reflect a DNA-cleaved complex in a rotationally rigid/frozen state or perhaps a complex in which one or both *hix* sites had re-ligated. In some cases, one or both of the DNA molecules broke during the course of the decatenation reaction, preventing further analysis. The general behavior of the complexes after complete relaxation was that they remained in the synapsed and cleaved state, where they were able to relax additional Ca inserted into the braid.

### Wild-type Hin is unable to promote braid relaxation

We tested whether braiding of the two duplexes might localize the Hin-bound *hix* sites in a manner that mimics the proposed function of the recombinational enhancer and Fis cofactor^42^ and thus lead to an enhancer-independent reaction by wild-type Hin. Hin-wt was incubated with five different braids for a total of 5000 minutes without any detectable decatenation (Fig. 5H, rightmost bar). Thus, the presence of the hyperactivating mutation in Hin-H107Y appears to be essential for Fis/enhancer-independent braid relaxation.

## Discussion

We have examined the mechanism of DNA strand exchange by individual synaptic complexes assembled by the Hin recombinase, with the result that we have directly observed the rotation of subunits within the synaptic tetramer (Fig. 1A). Rotational events of a variable numbers of turns are frequently interrupted by long pauses, indicative of a controlled rotation mechanism that is intrinsic to the Hin enzyme. Our studies on single recombination complexes have been done using a mutant enzyme (Hin-H107Y) which, unlike the wild-type enzyme, does not require a complex synaptic structure or cofactors to catalyze DNA strand exchange. Hin-H107Y is able to efficiently catalyze recombination on long linear DNA molecules through simple random collision synapses (this paper and see^28^). Bulk experiments measuring topological changes introduced into circular DNA molecules upon DNA strand exchange demonstrate that the Hin-H107Y tends to promote processive DNA exchanges (subunit rotations), unlike the single-round exchanges primarily catalyzed by the Fis/enhancer-dependent WT reaction. The results from single-synapses and bulk experiments are thus entirely consistent with each other and provide new insights into intrinsic controls of the subunit rotation mechanism for DNA strand exchange that are likely generalizable to other serine recombinases.

The results of the two-molecule braid relaxation experiments provide strong evidence for rotation of protein subunits that are covalently linked to the cleaved DNA ends. Relaxation of intermolecule catenation is dependent upon double strand cleavages of both DNA duplexes and rotary exchange of DNA strands within the Hin synaptic complex. Hin-H107Y-mediated relaxation events involved from a half turn up to 10 full turns of DNA (Fig. 6A), and therefore an equivalent number of 360° turns of synapsed subunits. Decatenation by Hin-H107Y is slow relative to that measured in single-molecule experiments performed on other DNA relaxing enzymes such as topoisomerases and nickases (Fig. 6B,D). Under equivalent reaction and mechanical conditions, the Hin synapse rotates at less than half of the rate of the Bxb1 synapse, indicative of a higher degree of molecular friction for Hin relative to Bxb1 serine integrase^26^,^41^. Moreover, Bxb1 reactions relax all catenation links in one step, again suggesting a smaller rotational control than by Hin.

Bulk reactions by Hin-H107Y on circular supercoiled DNA also exhibit variable numbers of subunit rotations prior to ligation. This is most clearly demonstrated in Fis/enhancer-activated reactions where DNA knots containing from 3 to >13 nodes, which would be derived from 1 to >6 360° turns of DNA, are generated from individual bulk reactions (Fig. 3). The distribution of knotted products is thus similar to the broad distribution of rotations observed in the braid decatenation experiments. Hin-H107Y reactions without Fis on supercoiled DNA substrates also generate knotted products indicative of iterative DNA exchanges, though these reactions exhibit a more complex product profile due to the substantial number of random collision synapses trapping variable numbers of DNA supercoils. Surprisingly, a majority of Fis/enhancer-independent reactions by Hin-H107Y proceed through a synaptic complex trapping a DNA branch like the WT enzyme; the Gin-M114V hyperactive mutant was also reported to catalyze most Fis/enhancer-independent reactions from a branched DNA synapse^43^.

By contrast, standard Hin-wt reactions overwhelmingly undergo just one subunit rotation before ligating in the recombinant DNA orientation to generate unknotted products, although multiple rotations can occur (e.g., Fig. 3A lane 3, 9 and 10^12^,^14^,^24^,^42^). The tyrosine mutant side chain within Hin-H107Y, which is predicted to stabilize rotating subunit pairs (Fig. 1C)^29^, is probably responsible for the increased processivity of subunit rotation by the hyperactive enzyme.

**Figure 2:**
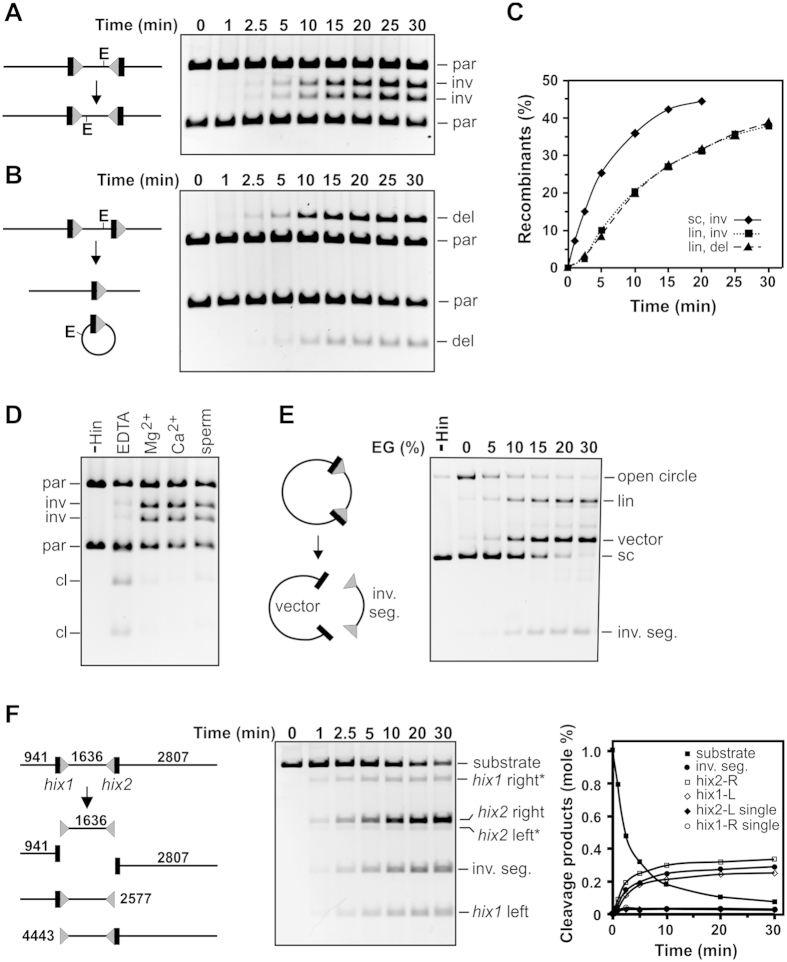
Properties of Hin-H107Y bulk reactions. (**A**) Inversion reaction on linear pMS634 containing *hixL* sites in inverted orientation. Digestion with EcoRV (E) was used to distinguish the orientation of the invertible segment: parental orientation (par), inverted orientation (inv). (**B**) Deletion reaction on linear pRJ858 containing *hixL* sites in direct repeat orientation. Digestion with EcoRV linearizes the deletion circle product (del). The starting pMS634 and pRJ858 substrates were linearized at their Nde I site; the *hix* sites are separated by 1636 and 1634 bp, respectively. (**C**) Plot of recombinants versus time from the reactions in A and B. An inversion reaction on supercoiled pMS634 is also included. **(D**) Hin-H107Y reactions (15 min) on supercoiled pMS634 with different metals or polyamines. Hin reactions contained 5 mM EDTA, MgCl_2_, CaCl_2_, or spermidine as denoted and were then digested with Nde I + EcoRV. cl denotes DNA molecules cleaved at the *hix* sites by Hin. (**E**) Hin-H107Y reactions (10 min) on supercoiled pMS634 in the presence of 5 mM EDTA and increasing concentrations of ethylene glycol (EG). Hin-catalyzed cleavage at both *hix* sites releases the invertible segment from the vector backbone; cleavage at one site linearizes (lin) the plasmid. **(F**) Cleavage time course reactions on pMS634 linearized at its Bsa I site. The reaction products are diagramed on the left; *hix1* right* and *hix2* left* are 4443 bp and 2577 bp fragments generated from single cleavages at *hix1* or *hix2*, respectively. Reaction products are quantified on the graph. A similar time course reaction on linear pRJ2421, used in the single-DNA molecule looping reactions, is given in Supplementary Fig. 2. Reactions in E and F were quenched by SDS.

**Figure 3:**
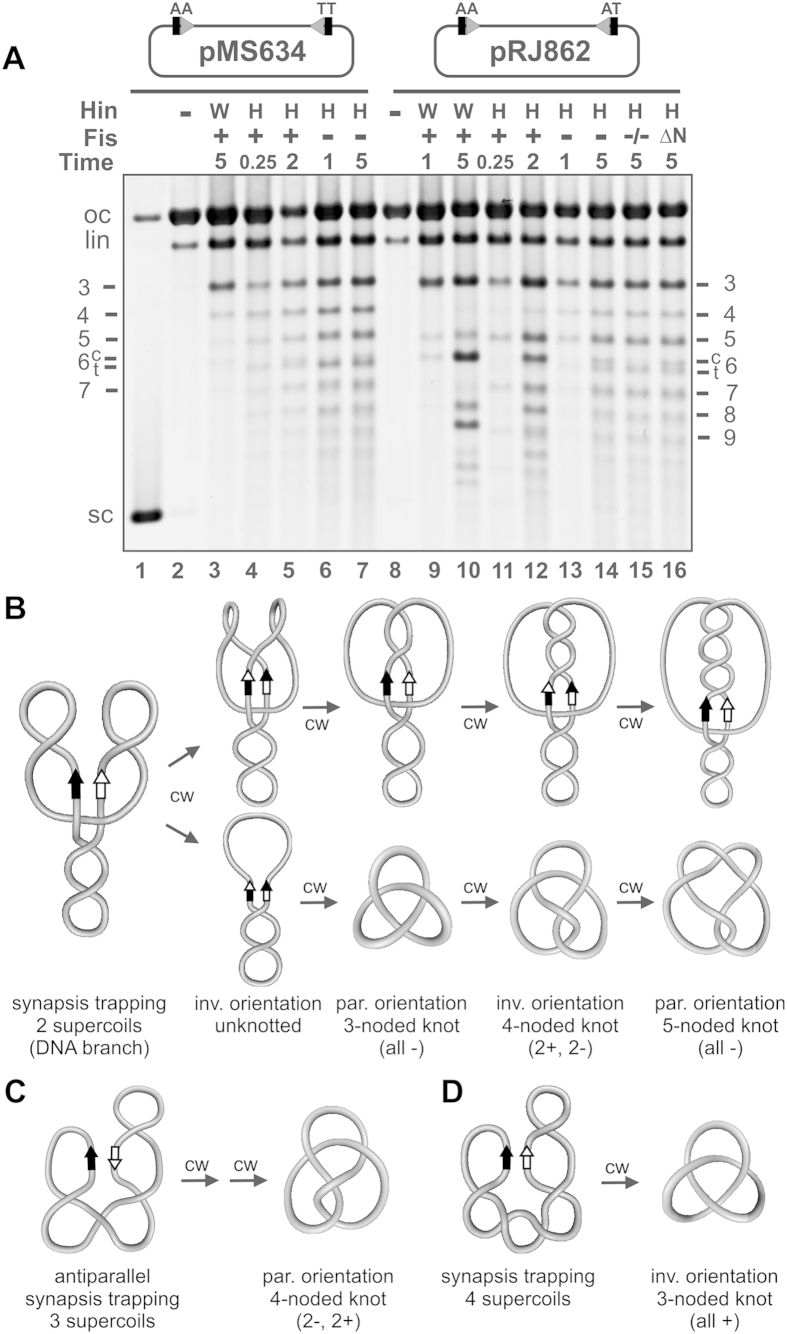
Topological analysis of reaction products generated by Hin-H107Y. (**A**) DNA knotting reactions on pMS634 (*hix-wt* sites) and pRJ862 (*hix-wt* + *hix-AT*) by Hin-wt (W) and Hin-H107Y (M) in the presence and absence of Fis as designated. Hin reaction times are in minutes. Hin reaction products were nicked with DNase I and subjected to agarose gel electrophoresis. The number of nodes in each knot band is given on the side (see^14^ for Hin-wt knot assignments); for 6-noded knots, compound (c) and twist (t) knots are resolved. oc, lin, sc refer to the open circular, linear and supercoiled species, respectively. All Hin reactions, except in lane 15 (indicated by “−/−”), included HU. The reaction in lane 16 contained the mutant Fis Δ(2–26) that binds DNA normally but is missing the N-terminal β-hairpin arms that interact with Hin. (**B**) DNA strand exchange from synaptic complexes trapping a supercoil branch (2 (−) nodes; referred to as a −2 synapse). This starting DNA geometry occurs in most Fis/enhancer-activated reactions and a substantial number of Fis/enhancer-independent reactions. Top panels are schematic representations of the DNA following one to four processive clockwise (cw) subunit rotations initiated from the −2 synaptic complex, and the bottom panels depict the product structures after resolution. Whereas reactions on pMS634 can ligate in the parental (par) or inverted (inv) orientation, reactions with pRJ862 can only ligate in the parental orientation after multiples of two subunit rotations. See also Supplementary Table 1. (**C**) Synapsis trapping 3 (−) supercoils where the *hix* sites are in an antiparallel orientation. Multiples of two subunit rotations are required in order to achieve base pairing and ligation. Two clockwise rotations generate a diagnostic 4-noded knot in the parental orientation as observed for Hin-H107Y reactions on pRJ862. (**D**) Synapsis trapping 4 (−) supercoils. A single clockwise rotation will generate a diagnostic trefoil with inversion, which has been confirmed for Hin-H107Y reactions on pMS634.

**Figure 4:**
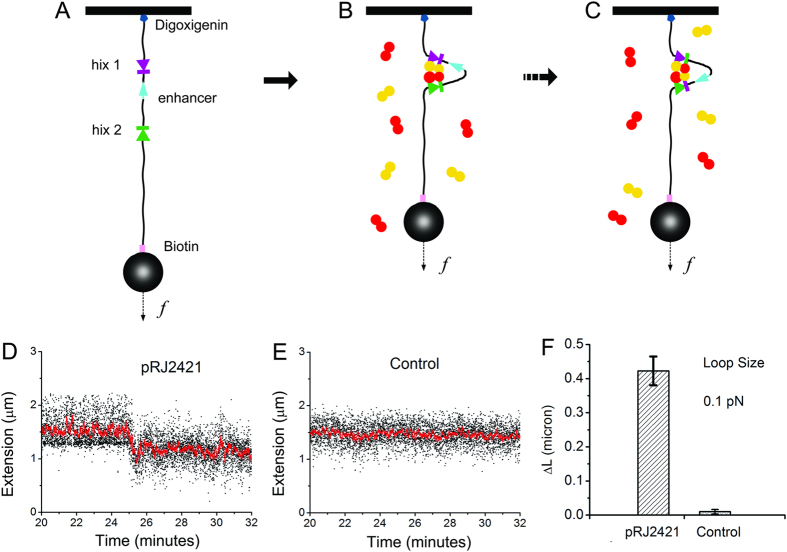
Recombination site (*hix*) synapsis on tethered single-DNA molecules by Hin-H107Y. (**A**) Linearized pRJ2421 was attached to the cover glass of a flow cell and to a streptavidin-coated paramagnetic bead on the opposite end. (**B**) Formation of the Hin synaptic tetramer generates a 2368 bp loop between the two *hix* sites. (C) Subunit rotation within the synaptic complex would not alter the position of the bead. (**D**) Hin-H107Y was introduced into the flow cell, and for this molecule a decrease of ~0.4 μm at 0.1 pN applied force occurred after 25 minutes, indicating formation of the synaptic complex; red line is filtered at 0.25 Hz. The waiting time for loop formation ranged from one to 250 minutes. Increasing the force to 2 pN opened the loops within 11 minutes. (**E**) Length of a representative control DNA (pFOS1 fragment) tether remained unchanged after Hin-H107Y addition. (**F**) Average length change (ΔL) of extensions at 0.1 pN determined from 5 experiments on pRJ2421 and on 6 kb pFOS1 control DNA fragment. Bars represent standard error.

**Figure 5:**
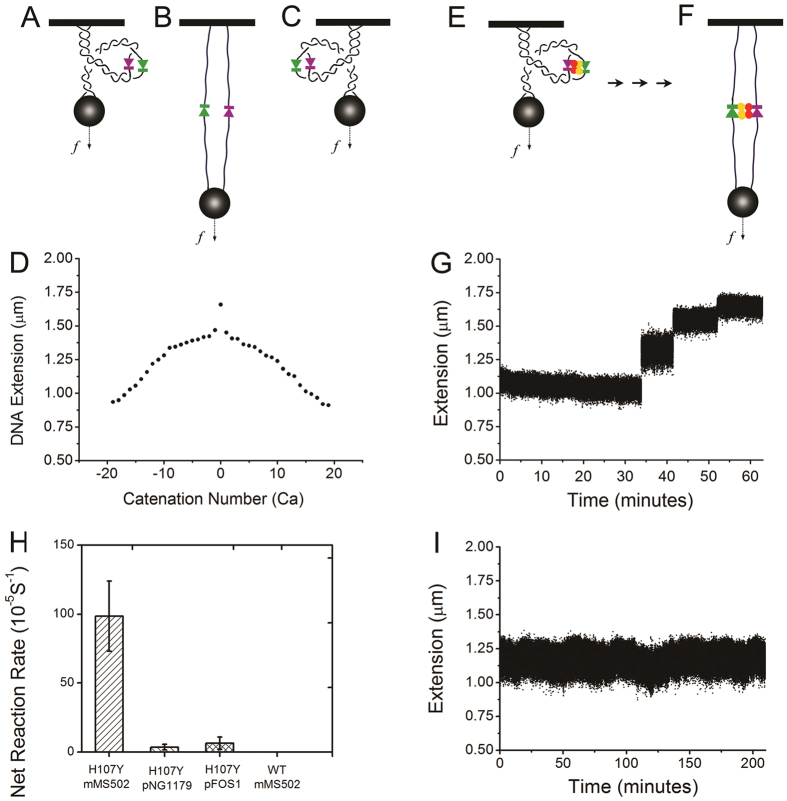
Relaxation of DNA braids by Hin-H107Y. (**A–C**) A pair of negatively braided, parallel, and positively braided double DNA tethers (solid black lines indicate double-stranded DNAs). (**D**) Extension of a double tether as a function of Ca (number of DNA interwinds and 360° bead turns relative to the unlinked, parallel DNA case), for 0.5 pN force in Hin reaction buffer. (**E**) Braid with *hix* sites in an initial synaptic complex after addition of Hin-H107Y. (**F**) Braid after complete relaxation by Hin-H107 protein. (**G**) Braid extension increased in a stepwise fashion with different size jumps during incubation with Hin-H107Y. (**H**) Net reaction rates for 4 types of DNA braiding experiments. For Hin-H107Y on mMS502 braids, data was obtained from 15 independent experiments with an average rate of (9.7 ± 2.5) × 10^−4^ s^−1^ for the first relaxation event. As expected, rates for Hin-H107Y reactions on pNG1179, or on the pFOS1 fragment, which do not contain *hix* sites, were much lower. No reaction was observed and the tethers remained intact for WT Hin and mMS502 during a total of 5000 minutes incubation on 5 braids. (**I**) Control DNA braid without *hix* sites (pNG1179) remained unchanged during a 200 minute incubation with Hin-H107Y.

**Figure 6:**
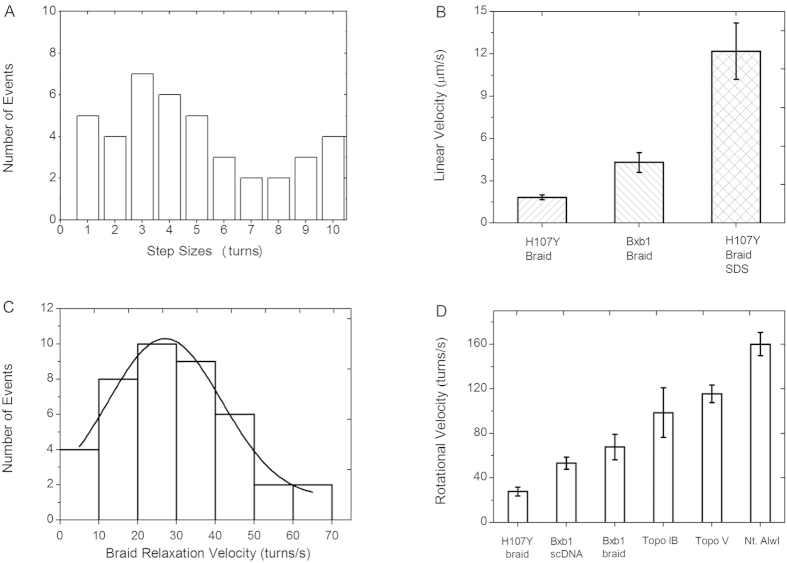
Event sizes and velocities for Hin-mediated braid relaxation. (**A**) Distribution of relaxation event sizes. The number of events as a function of full rotations (360°) is plotted. The mean of the rotation distribution is 4.9 turns. (**B**) Comparison of linear velocities of Hin-H107Y braid relaxation (left bar) with release of the bead by SDS-triggered dissociation of Hin-H107Y DNA-cleaved complexes (right bar). Bead velocity for Bxb1 integrase (middle bar^26^) is shown for comparison. (**C**) Rotational velocities from the 41 analyzed relaxation steps by Hin-H107Y obeyed a Gaussian distribution with a mean of 27 turns/s and a SD of 14.6; the maximum velocity observed was 69.4 turns/s. **(D**) Comparison of mean rotational velocities: Hin-H107Y braid relaxation, Bxb1 serine integrase supercoil relaxation, Bxb1 serine integrase braid relaxation, Topo IB supercoil relaxation, Topo IV supercoil relaxation, Nt. AlwI supercoil relaxation (data for enzymes other than Hin from^26^).

Most rotation reactions by Hin-H107Y were interrupted by pauses that lasted from <1 to up to 80 minutes in duration. Multiple lines of evidence show that these pauses are not coupled to ligation of DNA ends: (1) the reaction conditions used in the single molecule experiments inhibit the ligation step by Hin-H107Y (Fig. 2D–F and Supplementary Fig. 2); (2) Unlike Hin-wt^44^, Hin-H107Y does not support efficient ligation even when DNA cleaved intermediates generated from the EDTA+EG reaction conditions are switched to +Mg^2+^ and low EG conditions (see Supplementary Fig. 5), and (3) SDS denaturation of Hin during a pause in the rotation reaction on braids resulted in release of the magnetic bead in all tested cases. The latter result directly indicates that both DNA molecules are in a cleaved state at the time of denaturation. Presumably, pausing stalls the rotation in a conformer that is poised for the subsequent conformational and chemical steps of ligation, which is suppressed by our reaction conditions. After full relaxation of all intermolecular catenanes most of the braids remained in a synapsed, open (rotating), DNA-cleaved state.

The rather large rotational friction along with complete stalling of the rotation reaction provides evidence for a ratchet-like mechanism controlling subunit rotation. Although crystal structures show that the rotating surfaces of serine recombinases are remarkably flat and exclusively hydrophobic, they are not completely smooth^21^,^22^,^23^. DNA invertases and resolvases have hydrophobic residues that stack or interdigitate with each other across the rotating dimer interface. Substitutions of these residues in Hin lead to severe ligation defects, which can be explained by a failure to halt rotation at the ligation-competent conformer^45^. Stabilization of the Hin tetramer by H107Y mutation, combined with the use of reaction conditions preventing ligation, results in the rotations-pause-rotations profiles observed in the two-molecule braid relaxation experiments.

Topological studies on other serine recombinases have also suggested rotational “gating” mechanisms controlling the DNA exchange reaction (e.g., Gin DNA invertase^10^, Tn3 resolvase^11^,^13^, Sin resolvase^46^, ϕC31 integrase^47^, and Bxb1 integrase (under certain conditions^47^). For the Hin and Gin DNA invertases, the Fis/enhancer regulatory element provides an additional control over the number of subunit rotations because of the short DNA loop between *hixL* and the enhancer in the invertasome complex^12^,^14^,^24^. Uncontrolled rotational relaxation would be expected to lead to catastrophic consequences *in vivo*, such as global relaxation of chromosomal supercoiling and generation of DNA entanglements.

Finally we note that formation of a stable synaptic complex is not temporally coupled to DNA cleavage. Hin-H107Y synaptic complexes formed between two *hix* sites on the same tethered DNA molecule could be pulled apart, typically within a minute, by few-pN forces to generate full-length (uncleaved) DNA substrates. This suggests that the DNA cleavage process following synapsis is slow. Like ligation, cleavage of DNA strands is expected to involve additional conformational steps within the synaptic complex to position the active site serines proximal to the labile phosphodiester bond, although the nature of those conformational changes remains unknown.

## Methods

### DNA substrates

Plasmids pMS634 (inverted orientation *hixL* sites^48^), pRJ858 (directly repeated *hixL* sites^49^), and pRJ862 (inverted *hixL* sites with a mutation in the *hixL2* core nucleotides^14^) are schematically depicted in Supplementary Fig. 1; they were linearized by cleavage at their unique Nde I site. pRJ2421 was constructed from pMS618^48^ by creating unique Sac I and Nco I sites 50 bp apart within the backbone and inserting a 1.9 kb EcoR1 fragment containing the *lacI* gene into the EcoR1 site in the vector backbone to increase plasmid size. A 768 bp PCR-generated biotin-16-dUTP (Roche) labeled fragment was ligated onto the Nco I end and a 838 bp PCR-generated digoxigenin-11-dUTP labeled fragment was ligated onto the Sac I end to create the substrate used for single-DNA molecule synapsis assays (Supplementary Fig. 1). DNA braids were generated from DNA molecules amplified from mMS502, which contains the 26 bp *hixL* sequence inserted into the Sma I site of mp8^48^, using a 5′-biotin-labeled primer beginning 2931 nt from the *hixL* center and a 5′-digoxigenin-labeled reverse strand primer beginning 3152 on the other side of the *hixL* center (Supplementary Fig. 1). Control experiments used 6 kb linear fragments derived from non-*hix*-containing plasmids pFOS1 and pNG1179 (a derivative of pFOS1), via PCR reactions using biotin- and digoxigenin-labeled primers as described^26^.

### Bulk Hin reactions

Unless otherwise noted, Hin-H107Y recombination reactions were performed in 20 mM Hepes (pH 7.5), 80–100 mM NaCl, 5 mM MgCl_2_, 4 mM CHAPS, 1 mM DTT, 200 μg/ml polycytidylic acid, 15% ethylene glycol (EG), 2 nM (0.05 pmol) plasmid DNA substrate and 25–50 nM Hin. Reactions were typically quenched with 1/10 volume of 1% diethylpyrocarbonate, then incubated at 65° for 30 min to remove the DEPC, and finally digested with restriction enzymes to distinguish the recombinant products. For the knotting reactions shown in this report, plasmid nicking after the Hin reaction was performed using DNase I in the presence of 400 μg/ml ethidium bromide at room temperature for 20 min; some linear DNA is generated by this method. Samples were extracted with phenol: chloroform before loading on a 0.85% agarose gel in Tris-Phosphate-EDTA buffer^50^. DNA cleavage reactions were performed in the same buffer except 5 mM EDTA was substituted for MgCl_2_, and 30% EG was added; the reactions were quenched with 1% SDS followed by digestion with proteinase K^44^. Band intensities were quantified using ImageQuant (GE Healthcare Life Sciences). Hin was purified as described^29^.

### Single-DNA looping experiments with magnetic tweezers

pRJ2421 was tethered to streptavidin-coated 2.8 μm diameter paramagnetic beads (Dynabeads, Invitrogen) in phosphate-buffered saline (PBS) buffer containing 0.5 mg/ml bovine serum albumin (BSA) and mounted in a flow cell on a magnetic tweezers setup as described^40^,^51^. The extension-force relationship expected for a single-DNA molecule^39^ was used to check that one DNA molecule was tethering the bead. After a single tether was confirmed, the PBS in the flow cell was substituted with Hin buffer that contained 20 mM Hepes (pH 7.5), 80 mM NaCl, 4 mM CHAPS, 5 mM EDTA, 90 ng/μl herring sperm DNA (hsDNA, Promega), and 30% EG. Then, 70 nM Hin-H107Y in Hin buffer was introduced. All single molecule experiments were performed at 37 °C using an objective temperature controller (20–20 Technology, Inc.). DNA extension was monitored by real-time analysis of the bead image at ~100 measurements per second.

Use of herring sperm DNA was essential to avoid nonspecific Hin-DNA interactions. In experiments with single DNAs without herring sperm DNA we found that 70 nM Hin-H107Y in Hin buffer compacted all DNAs (reduced their extension) by an amount of about 1 μm over a period of a few minutes. This deleterious effect was progressively reduced by 10 to 70 ng/μl hsDNA and eliminated by 90 ng/μl hsDNA.

### Two-DNA-braid decatenation experiments using magnetic tweezers

mMS502 fragment tethers were prepared as in the looping experiments. Beads tethered by two DNAs were identified by comparing their extension versus force relationship with that expected for two DNAs. The tether was then further tested by determining the extension versus Ca (catenation number) relationship^26^,^52^ at a fixed 0.5 pN force; measurement of this relation provides a check that the bead is tethered by two DNAs, and also provides calibration data to convert extension measurements into catenation number. After calibration, the tether was twisted to Ca = −10 to form a braid. The working buffer and protein solution was subsequently introduced to the sample. After complete braid relaxation was observed, Hin buffer containing 2% SDS was introduced into the sample. In some experiments the SDS solution was added after the first braid decatenation event.

### Ethics statement

All DNA segments used in this work were of bacterial origin. All experimental methods and protocols were approved by, and carried out in accordance with guidelines approved by, the Northwestern University Office for Research Safety, and by the University of California at Los Angeles Environment, Health & Safety Office.

## Additional Information

**How to cite this article**: Xiao, B. *et al*. Controlled rotation mechanism of DNA strand exchange by the Hin serine recombinase. *Sci. Rep*. **6**, 23697; doi: 10.1038/srep23697 (2016).

## Supplementary Material

Supplementary Information
