# Supplementary material for: Controlled rotation mechanism of DNA strand exchange by the Hin serine recombinase
Source: Sci Rep. 2016 Apr 1;6:23697. doi: 10.1038/srep23697 (PMC4817059; doi:10.1038/srep23697)
Supplement: Supplementary Information [file srep23697-s1.pdf]

Supplementary Information for

Controlled rotation mechanism of DNA strand exchange by the Hin serine recombinase

Botao Xiao [1,2,3], Meghan M. McLean [4], Xianbin Lei [1], John F. Marko [2,5†], Reid C. Johnson [4]*

[1] School of Physics, Huazhong University of Science and Technology, Wuhan, Hubei 430074, China

[2] Department of Physics and Astronomy, Northwestern University, Evanston IL 60208

[3] Key Laboratory of Molecular Biophysics of Ministry of Education, Huazhong University of Science and Technology, Wuhan, Hubei 430074, China

[4] Department of Biological Chemistry, David Geffen School of Medicine at UCLA, Los Angeles CA 90095-1737

[5] Department of Molecular Biosciences, Northwestern University, Evanston IL 60208

*Corresponding author, rcjohnson@mednet.ucla.edu

†Co-corresponding author, john-marko@northwestern.edu

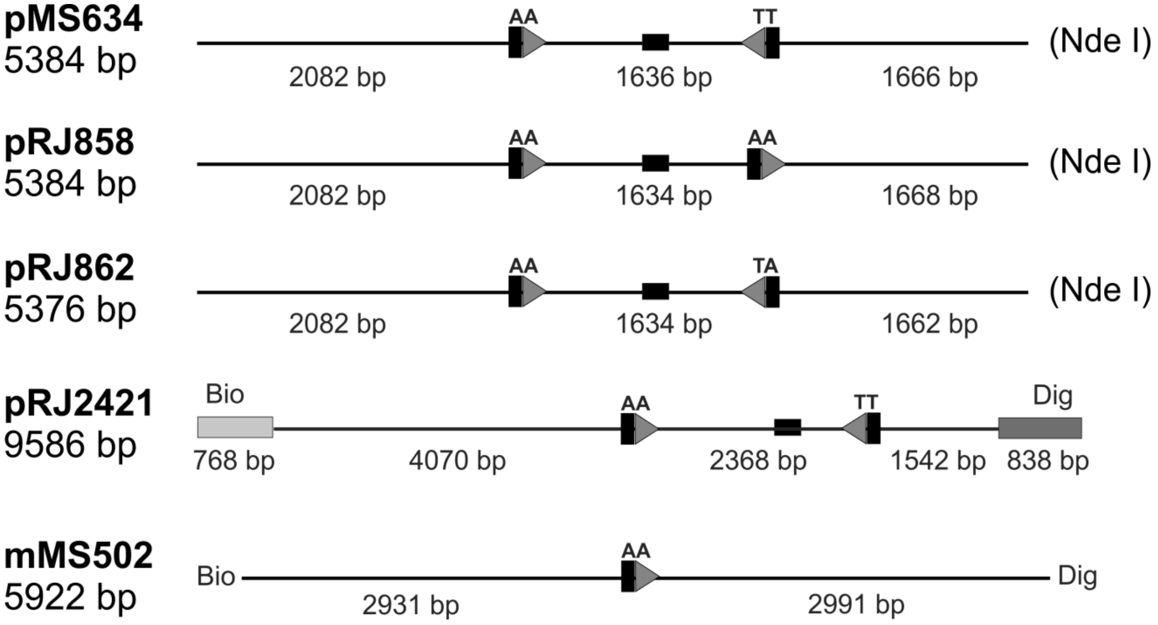

Supplementary Figure 1. Hin DNA substrates. The Hin recombination sites *hixL* are shown in their relative orientations as black-grey triangles, and the recombinational enhancer, when present, is depicted as a black rectangle. The two core nucleotides at the *hixL* center where the staggered DNA cleavage-ligation occurs are given: (AA)/(TT) is the wild-type *hixL* sequence and (AT)/(TA) is a mutant *hix* site that cannot be ligated after an odd number of subunit rotations when paired with a wild-type *hix* site as in pRJ862. The lengths (in bp) of the DNA molecules used in the experiments are given below the plasmid or M13 phage (mMS502) names.

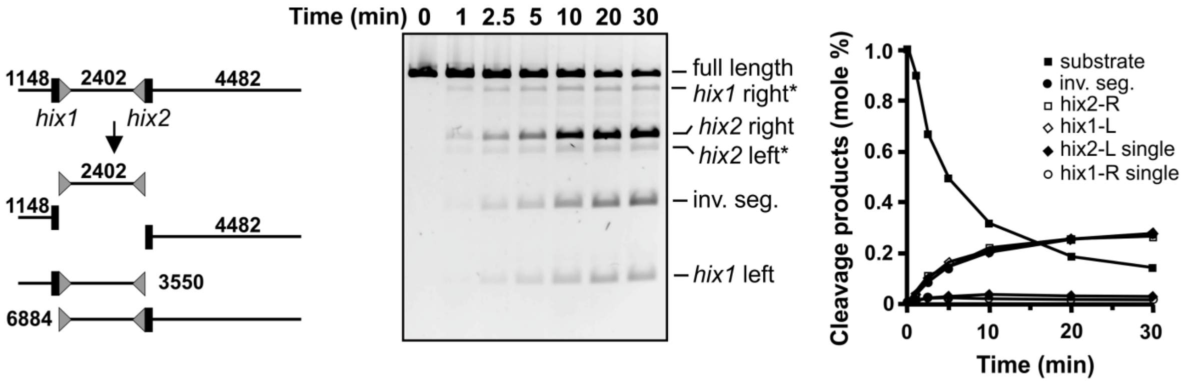

Supplementary Figure 2. Hin-H107Y time course reaction on linear pRJ2421 used in the single-DNA molecule looping reactions. Reaction conditions are identical to those in Fig. 2F. pRJ2421 was linearized at its unique MluI site.

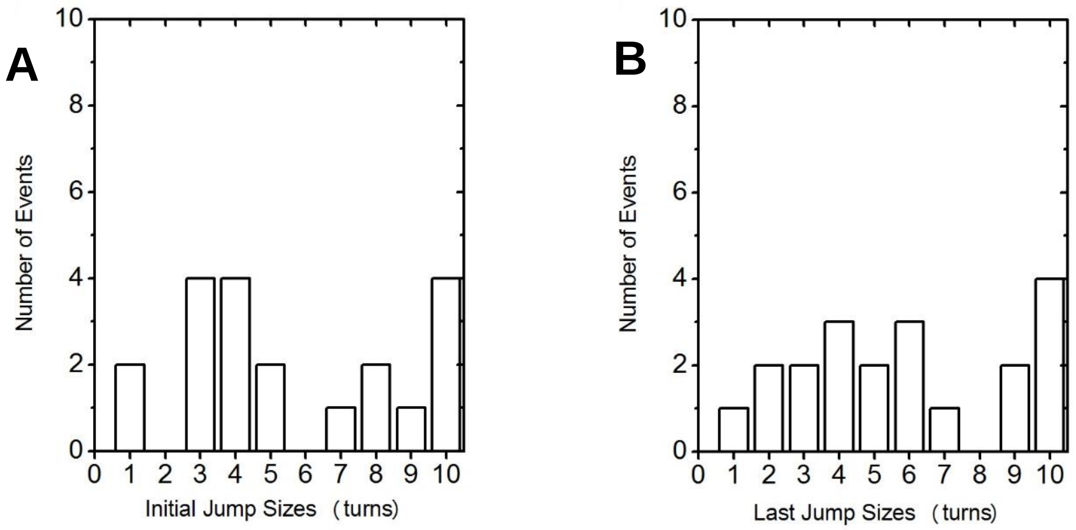

Supplementary Figure 3. Plots of the number of rotations (turns) occurring at the first relaxation event (A) and the last relaxation event (B). Four of the braided molecules were relaxed completely from the starting Ca = -10.

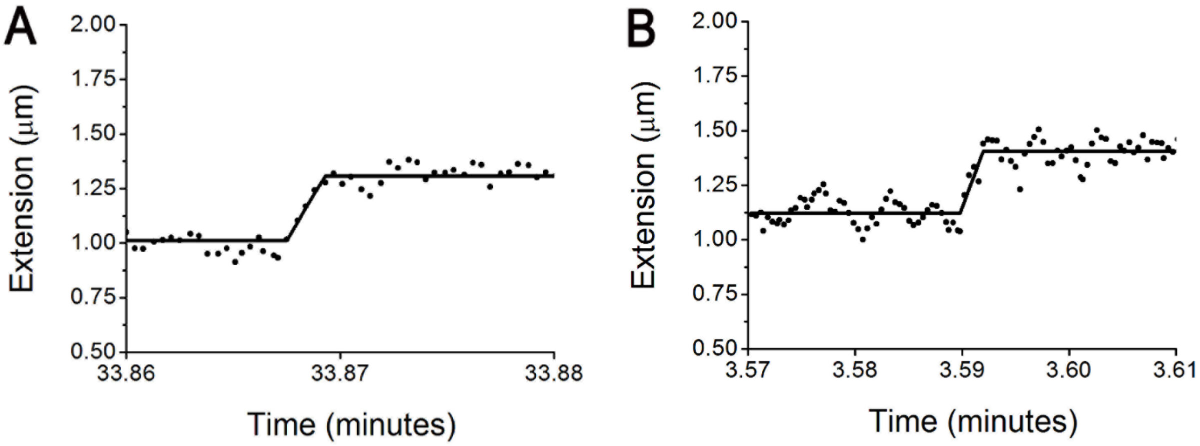

Supplementary Figure 4. Magnified relaxation events exhibited by two independent braided mMS502 DNAs with 0.5 pN tension, showing two events with ~5 rotations. Fit slopes (A: 2.1±0.2 μm/s; B: 2.2±0.5 μm/s) were converted to rotational velocities via calibration data as in Fig. 5D.

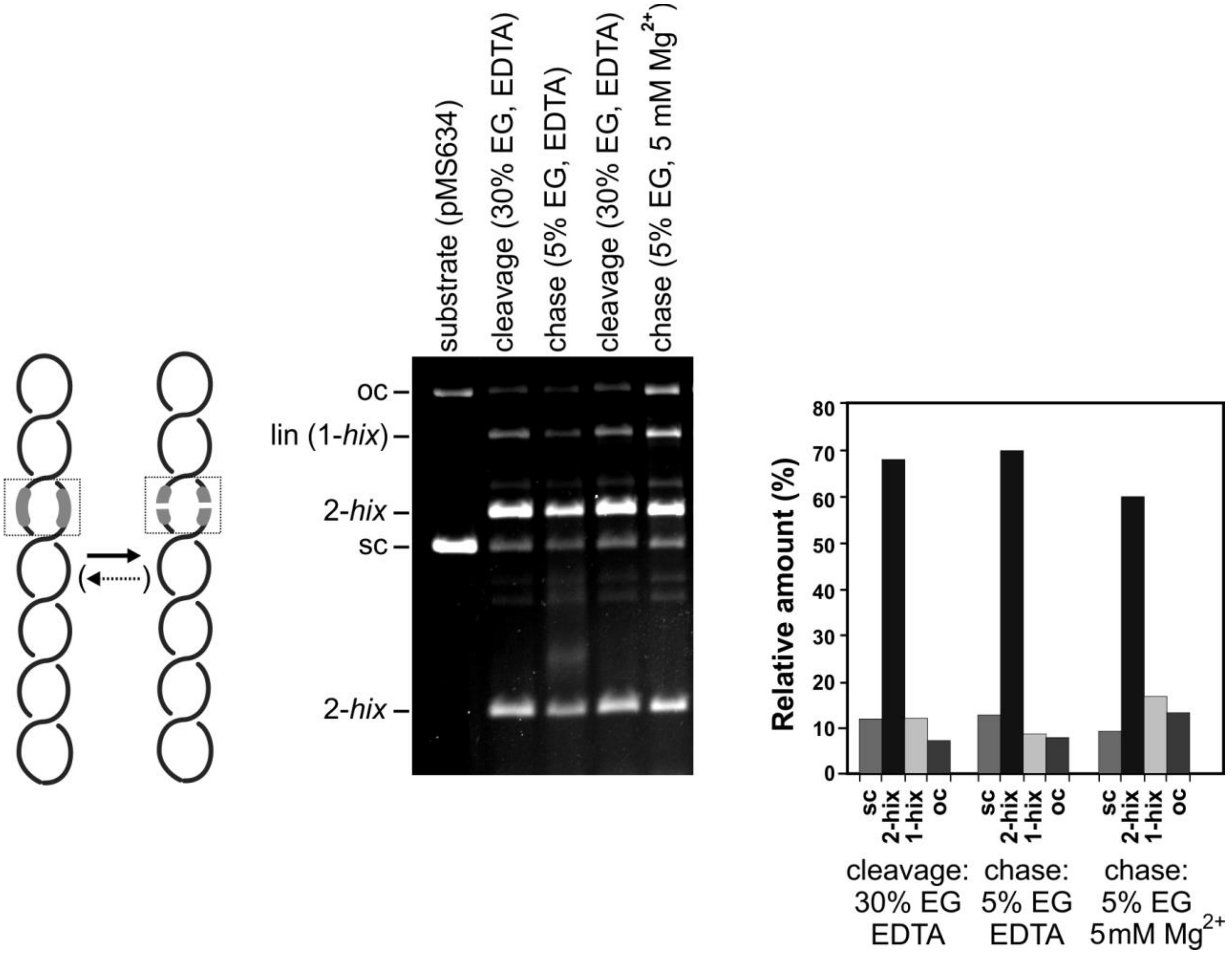

Supplementary Figure 5. Hin-H107Y cleavage/chase/ligation experiments demonstrating extremely inefficient ligation from a stable cleavage complex formed in 30% EG plus EDTA even when EG is then lowered to 5% and Mg^{2+} is added.

Number of (-) nodes trapped[1]	*hix* site orientation[2]	Number & direction of subunit rotations[3]	Number & chirality of knot nodes[4]
0	par	cw or ccw	no change
1	anti-par	2-cw<br>4-cw<br>6-cw<br>2-ccw<br>4-ccw	loss of 3 sc<br>3: all +<br>5: all + (torus)<br>3: all –<br>5: all – (torus)
2	par	2-cw<br>4-cw<br>6-cw	3: all – (see Fig. 3B)<br>5: all – (see Fig. 3B)<br>7: all –
3	anti-par	2-cw<br>2-ccw	4: 2+ 2- (see Fig. 3C)<br>5: all –
4	par	2-cw<br>2-ccw	5: all –<br>6: 2+ 4-
5	anti-par	2-cw<br>2-ccw	6: 2+ 4-<br>7: all –
6	par	2-cw<br>2-ccw	7: all –<br>8: 2+, 6-

Supplementary Table 1. Formation of knotted DNAs on pRJ862 (*hixL-wt* x *hixL-AT*)

[1] Number of DNA nodes trapped on (-) supercoiled circular DNA by Hin synapsis.

[2] Because the *hix* sites in pRJ862 are in inverted orientation, synapses with 2-, 4- and 6-nodes trapped between *hix* sites have the sites in a parallel (par) orientation whereas synapses with 3- and 5- trapped nodes have the sites in an antiparallel (anti-par) orientation.

[3] In all cases even numbers of subunit rotations in the same direction are required for ligation. For the -2 (branched) intermediate, only the products of clockwise (cw) rotations are listed because the negative supercoiling energy is uniquely transduced into cw rotations. In the cases where 3 to 6 supercoils are trapped between synapsed *hix* sites, the products of 2 cw or 2 counterclockwise (ccw) rotations are given because both are expected to occur.

[4] The number followed by the predicted chirality of the nodes is given. All >3-noded knots listed are of the twist family unless noted (see N.J. Crisona *et al.* 1994, J. Mol. Biol. 243, 437-457).
